# Metastasis: Recent Discoveries and Novel Perioperative Treatment Strategies with Particular Interest in the Hemostatic Compound Desmopressin

**DOI:** 10.2174/138920111798377076

**Published:** 2011-11

**Authors:** D.F Alonso, G.V Ripoll, J Garona, N.B Iannucci, D.E Gomez

**Affiliations:** 1 Laboratory of Molecular Oncology, Quilmes National University; 2 School of Pharmacy and Biochemistry, University of Buenos Aires and Romikin S.A.; Buenos Aires, Argentina

**Keywords:** Angiogenesis, breast cancer, desmopressin, hemostasis, Surgery, tumor spread, vasopressin analog.

## Abstract

Metastatic disease is responsible for most of cancer lethality. A main obstacle for therapy of advanced cancers is that the outcome of metastasis depends on a complex interplay between malignant and host cells. The perioperative period represents an underutilized window of opportunity for cancer treatment where tumor-host interactions can be modulated, reducing the risk of local recurrences and distant metastases. Blood-saving agents are attractive compounds to be administered during tumor surgery. Desmopressin (DDAVP) is a safe and convenient hemostatic peptide with proved antimetastastic properties in experimental models and veterinary clinical trials. The compound seems to induce a dual angiostatic and antimetastatic effect, breaking the cooperative function of cancer cells and endothelial cells during residual tumor progression. DDAVP is therefore an interesting lead compound to develop novel synthetic peptide analogs with enhanced antitumor properties.

## INTRODUCTION

Metastasis is formed when a cell, or a group of cells, leaves the original site of the primary tumor and establishes a new colony in a distant site. To form an overt metastasis tumor cells must overcome the physical, physiological and immunological constraints imposed by the tissue milieu and initiate invasive growth [1]. Traditionally, cancer progression is viewed as a complex multi-step process, where tumor cells gradually acquire the capacity to invade and intravasate into the systemic circulation or the lymphatic system. However, successful colonization at distant organs depends on the ability of circulating tumor cells to survive in the blood stream, to leave circulation by extravasation and then to adapt to the new microenvironment [2].

Metastatic disease is responsible for most of cancer lethality, therefore understanding the intricate interplay between tumor cells, soluble factors, extracellular matrix (ECM) and host cells during cancer spread is crucial to design successful therapeutic interventions. Emerging evidence indicates that disseminated tumor cells are present in the circulation in large numbers even at early stages of cancer, before metastatic foci at distant organs can be detected [3]. Furthermore, it has been suggested that solid tumor surgery can promote tumor growth, and also facilitate metastatic dispersal and distant growth [4]. In addition, a recent discovery suggests that circulating tumor cells are able to reinfiltrate tumors at their primary organs and promote tumor progression [5]. This intriguing phenomenon of “tumor-self seeding” provides new insights into the dynamics of tumor progression. Each metastatic site may serve as a nesting ground to generate tumor variants that repopulate other metastatic sites as well as the primary tumor [6].

## BIOLOGY OF TUMOR INVASION AND METASTASIS

Tumor invasion can be classically divided into three sequential steps: adhesion of tumor cells to the basement membrane and ECM, disruption of matrices by proteolytic digestion, and migration through the digested barrier [7]. Adhesion of tumor cells to the basement membrane involves specific anchoring glycoproteins, such as fibronectin, laminin and collagens, which bind to a variety of tumor cell surface receptors. To penetrate the ECM, invading cells must disrupt local segments in the organized structure of the basement membrane, a tightly regulated process involving proteolytic enzymes [8]. Metastatic capacity also depends on angiogenesis, a process by which the tumor induces the formation of new blood vessels, beginning with capillary buds and progressing to a vascular network. Metastasis requires active cell motility, not only for the endothelial cells during angiogenesis but also for the tumor cells. The collective migration of cells as a cohesive group is a hallmark of the tissue remodelling events during embryogenesis, wound repair and cancer invasion. Migratory cells move as sheets, strands or clusters rather than individually, and use similar actin- and myosin-mediated protrusions and guidance by extrinsic chemotactic and mechanical cues as used by single cells [9]. The newly formed blood vessels within and around the tumor mass provide nutrients for tumor growth, and also create an easy access to circulation for metastatic spread [10]. Interestingly, a biological similitude between tumor invasion and angiogenesis underlines a cooperative function of cancer cells and endothelial cells during tumor progression.

Although a large number of cancer cells can be released from primary tumors into the circulation, less than 0.1% will survive to produce metastatic tumors [11]. This so-called “metastatic inefficiency” has been well-documented in experimental animal models. As a result of interactions with the microvasculature at the target organ, some malignant cells are eliminated instantly by mechanical trauma where others are killed relatively slowly, over minutes or hours, by the inflammatory and immune responses [12, 13].

Metastatic cells entering into the blood stream possess the capacity to interact with all components of the hemostatic system, such as platelets, effectors of coagulation and fibrinolysis, leukocytes and the vascular endothelium. These interactions may determine the formation of microthrombi that increase the efficiency of the metastatic cascade [14]. Seminal experimental studies demonstrated that fibrin deposition enhances intravascular tumor cell aggregation and trapping in the target organ, and also protects circulating cells from destruction by host immunity [15]. In the last years research studies have greatly improved our knowledge on the complex bidirectional relationship of cancer and hemostasis. Understanding the molecular basis of the underlying mechanisms may help to pharmacologically modulate the hemostatic properties of malignant cells to interfere with the progression of the disease [14, 16].

## THE PERIOPERATIVE “WINDOW OF OPPORTUNITY”

Excisional surgery is the primary treatment modality for solid cancers. However, many common epithelial cancers carry a poor prognosis even after curative resection of early stage tumors. Tumor progression in these cases has been attributed to the persistence of occult neoplastic cells in various body compartments, as a sign of minimal residual disease (MRD). Regrettably, little is known about the conditions required for the persistence of micrometastatic dormancy or the escape from the dormant phase into the active phase of metastasis formation [17]. There is increasing evidence indicating that tumor removal alters the growth of MRD, leading to perioperative tumor growth. Mechanisms by which surgery could promote tumor growth and worsen prognosis include direct seeding of tumor at local sites, tumor manipulation, stimulation of subclinical tumor by postsurgical inflammation, and accelerated metastatic tumor growth due to loss of inhibitory factors derived from the primary tumor [4]. Distant metastases are kept in a dormant state by antiangiogenic mediators produced by the main tumor mass. Preclinical studies have shown that surgical excision of the primary cancer reactivates growth of metastasis [18, 19]. Furthermore, the inflammatory process associated with surgery shares mediators with tumor growth and invasion. Macrophages, fibroblasts and humoral factors of inflammation have been shown to enhance tumor growth in preclinical models [19, 20].

Coffey *et al.* have proposed a novel therapeutic paradigm, that the perioperative period “represents a window of opportunity during which the patient may be further protected against the oncological effects of tumor removal” [21]. Because neoplasia is a systemic disease, this concept may be relevant to all patients undergoing surgery for cancer. In the same line, Van der Bij *et al.* have recently analyzed the underlying mechanisms in colorectal cancer surgery and concluded that “perioperative therapeutic window of opportunity offers promising means of improving patient outcome, but is unfortunately underutilized” [22]. Surgical removal of the primary tumor provides the best chance of long-term disease-free survival for patients with solid cancers but, paradoxically, surgery itself contributes to the development of both local recurrences and distant metastases.

It has been suggested that surgical manipulation can provoke liberation of viable cancer cells. The presence of malignant cells in the peripheral blood has been confirmed by RT-PCR in patients undergoing breast cancer surgery [23]. Similarly, conventional chemotherapy may cause a mobilizing effect on breast cancer cells [24]. An immunohistochemical study on the pattern of sentinel lymph node metastasis in breast cancer suggested that the frequency of positive nodes is increased after instrumentation of the tumor site [25]. Other authors reported the histological findings in a series of axillary lymph node dissections taken approximately two weeks after breast biopsy. They described the presence of epithelial cells in the subcapsular sinus of draining lymph nodes that may be attributed to mechanical transport of tumor breast epithelium secondary to the previous needle or surgical manipulation [26]. It was also reported that surgical trauma can promote metastasis in gastrointestinal cancers. Esophageal cancer operation resulted in a significant increase of circulating tumor cells, as measured in blood samples by quantitative RT-PCR, resulting in further development of metastasis [27]. Many studies in colorectal cancer patients undergoing colonoscopy or endoscopic insertion of colonic stents demonstrated that mechanical forces cause liberation of cancer cells [28, 29].

Surgical trauma and concomitant wound-healing processes induce local and systemic changes, including impairment of tissue integrity and production of inflammatory mediators and angiogenic factors [22]. High concentrations of proangiogenic factors were detected in surgical wound fluid samples from breast cancer patients, suggesting that they may need to be antagonized using perioperative systemic or local therapy [30]. In this context, local and distant recurrence of breast cancer may be because of the perioperative stimulation of residual cancer cells [31]. Besides, surgery can lead to immune suppression that may predispose to tumor growth and spread [32].

A perioperative treatment may offer the opportunity to modulate the early wound environment and reduce locoregional cancer recurrence rates. Moreover, enhanced coagulation after tumor manipulation may contribute to a rapid encapsulation of residual tumor tissue, limiting intravasation of tumor cells. As shown in Table **1**, blood-saving agents, such as tranexamic acid [33], aprotinin [34] and desmopressin (DDAVP) [35], have been used during surgery. Perioperative and postoperative administration of the antifibrinolytic agent tranexamic acid reduced the frequency of wound complications in women with breast cancer undergoing lumpectomy or mastectomy [33]. Intraoperative infusion of the hemostatic agent aprotinin, a non-specific protease inhibitor, was associated with a significant survival benefit in patients who underwent liver resection for colorectal cancer metastasis [34].

## THE VASOPRESSIN ANALOG DDAVP

DDAVP (1-deamino-8-D-arginine vasopressin) is a synthetic analog of the antidiuretic hormone vasopressin, firstly described by Zaoral and coworkers in 1967 [36]. The peptide sequence of vasopressin includes 9 aminoacid residues, having a disulfide bridge between positions 1 and 6. The substitution of L-arginine for D-arginine in position 8 drastically reduces the pressor effect of DDAVP and cysteine deamination in position 1 prolongs its half-life and the other biological effects, as shown in Fig. (**1**). In contrast to vasopressin, which binds to different cell membrane receptors (V1a, V1b/V3 and V2), DDAVP is a selective agonist for the V2 membrane receptor [37]. This vasopressin receptor subtype is expressed in the kidney collecting duct, mediating the antidiuretic action, and is also present in endothelial cells, mediating most of the non-renal effects of DDAVP, including a potent hemostatic effect [35, 38].

DDAVP was initially used for antidiuretic replacement therapy in the management of diabetes insipidus and enuresis. Additionally, DDAVP is a well-tolerated and convenient hemostatic compound that can be used during surgery in a number of clinical conditions with bleeding diathesis [35]. It has several effects on the hemostatic and fibrinolytic system, causing release of von Willebrand factor (VWF), coagulation factor VIII and tissue-type plasminogen activator (tPA) from microvascular endothelial stores. VWF is a large glycoprotein playing a role in primary hemostasis, by mediating adhesion of platelets to the subendothelium. VWF is synthesized in endothelial cells and megakaryocytes, and stored as a multimer in specialized secretory granules named Weibel-Palade bodies [38, 39]. DDAVP-induced VWF secretion results from V2 receptor-mediated, cAMP-dependent exocytosis.

The presence of vasopressin receptors was reported in transformed epithelial cells, and also documented in several tumor variants, including breast and lung malignancies [40]. As indicated by North *et al.*, vasopressin gene-related expression is a feature of all breast cancers, and products of this expression are attractive as potential targets for therapy [41]. In addition, neuropeptide receptor expression was detected in a wide panel of human tumor cell lines [42].

DDAVP exhibited modest but significant antiproliferative effects on MCF-7 and Skbr3 V2 receptor-expressing human breast carcinoma cell lines [43, 44]. Such action was clearly mediated through agonist V2 receptor signaling, and thus involved activation of adenylate cyclase followed by intracellular cAMP elevation. The anti-mitogenic effect could be blocked by the selective V2 receptor antagonist SR121463 [43]. It was also reported that the natural hormone vasopressin inhibited the *in vitro* growth of MCF-7 human breast carcinoma cells at high concentrations [45].

Years ago, we have reported that DDAVP can modulate both cell growth and secretion of the serine protease urokinase in cultures of mouse mammary tumor cells [46]. More recently, preliminary results indicated that treatment of MCF-7 monolayers with DDAVP, in the presence of proper concentrations of plasminogen, induced the formation of angiostatin, a natural, tumor-born inhibitor of angiogenesis [47]. Since angiostatin is an internal fragment of plasminogen [48], it is likely that DDAVP induces a V2 receptor-dependent proteolytic processing leading to angiostatin formation.

## ANTIMETASTATIC EFFECTS OF DDAVP

We communicated for the first time that intravenous administration of DDAVP can impair development of distant metastasis. DDAVP, at doses of 1-2 μg/kg, inhibited by 70% the experimental lung colonization of aggressive mammary cancer cells [49] and dramatically decreased lymph node and distant metastasis in a mouse model of breast tumor manipulation and surgical excision [50]. We ruled out that DDAVP induces direct cytotoxicity on tumor cells in such animal model, indicating a complex biological mechanism underlying the antimetastatic properties of the compound. Antimetastatic resistance was obtained without overt toxic effects in mice [50].

Considering the antimetastatic properties of DDAVP, as well as its well known hemostatic effect and safety, the compound could be an excellent candidate for adjuvant therapy to breast tumor surgery. We conducted a pilot veterinary clinical trial in surgically treated bitches with locally-advanced mammary cancer [51]. DDAVP was administered in 2 doses, the first 30 minutes before and the second 24 hours after surgery, at a clinically-relevant hemostatic dose for dogs (1 μg/kg). Perioperative treatment significantly prolonged survival, having a beneficial effect both on disease-free interval and on overall survival. DDAVP appeared to be safe at this dose in canine cancer patients and decreased intraoperative bleeding [51]. An extended veterinary clinical trial recently confirmed these results, showing a particular survival benefit in patients with more aggressive carcinoma [52].

In comparison with other blood-saving agents such as tranexamic acid or aprotinin which display a nonspecific antifibrinolytic activity [33, 34], DDAVP seems to exert a specific effect on membrane receptors present in both tumor and endothelial cells. DDAVP improves perioperative hemostasis and may contribute to encapsulation of residual tumor tissue, limiting extravasation of metastatic cells. In mice, intravenous DDAVP prevented aggregation of mammary carcinoma cells, therefore reducing the efficiency of the metastatic process [49]. As mentioned above, intravenous injection of DDAVP induces a rapid release of multimeric forms of VWF from microvascular endothelial cells, reaching peak levels at about 60 minutes and having a plasma half-life of 8-10 hours [35]. Terraube *et al.* showed that VWF plays a protective role against tumor cell dissemination in a mouse model [53]. VWF might participate in the interaction of tumor cells with the subendothelium, and appears to obstruct metastasis by reducing sustained adherence of malignant cells in the microvasculature at the target organ. Furthermore, VWF was shown to directly induce apoptosis of tumor cells *in vitro* and caused death of metastatic cells arrested in the lungs [54]. Taking together these observations suggest that abrupt release of VWF from the microvasculature may favor the collapse of early metastatic foci. Thus, it is likely that DDAVP injection not only inhibits perioperative metastatic events but also combats spontaneous micrometastases that occurred before surgery.

## PERIOPERATIVE DDAVP IN HUMAN CANCER

Although no controlled clinical trials were conducted yet in humans to explore the new indication of DDAVP as a hemostatic adjuvant during tumor surgery, there are several reports that document its satisfactory perioperative use in cancer patients [55-59]. Mostly, they referred to patients with different hemostatic disorders in whom DDAVP administration prevented bleeding during excisional surgery.

One of the first reports showed the successful use of DDAVP in a woman with von Willebrand disease having an ovarian tumor [55]. In another case, a woman of 33 years with type II thrombasthenia received DDAVP during the resection of a breast tumor diagnosed as fibroadenoma. The compound was administered intravenously at a dose of 0.4 μg/kg, with good tolerance and hemostasis control. Bleeding time was reduced from 10 to 4 minutes, platelet adhesion on glass was increased from 1.8 to 37% and other hemostatic parameters were also increased, such as VWF multimers [56]. A patient of 67 years with von Willebrand disease received DDAVP during a major thoracic surgery for the resection of a lung cancer. Lobectomy and extirpation of lymph nodes were carried out and control of hemostasia was successful [57]. A more recent work reported the use of DDAVP in a patient with type A hemophilia that was subjected to a hepatic resection due to colon cancer metastasis [58]. As well, the use of DDAVP has been recommended in patients with type A hemophilia subjected to surgery to excise skin cancer lesions, in order to prevent the peri and postoperative bleeding [59].

## PERSPECTIVES

The major cause of death from cancer is due to metastatic spread of the disease, existing several factors that account for the failure to treat residual metastasis. Perhaps the greatest obstacle for therapy of an advanced cancer is that the outcome of metastasis depends on multiple “cross-talk” interactions of disseminated cells with homeostatic mechanisms which the tumor cells usurp [11]. The organ microenvironment can influence the response of metastases to therapy, thus treatment of metastasis should be targeted against both the malignant cells and critical homeostatic factors that promote metastasis [2, 11]. In this sense, the perioperative period is an attractive window of opportunity to modulate tumor-host interactions, and thus reduce the risk of local recurrences and distant metastases.

The biological effects of perioperative administration of DDAVP on both endothelial and V2 receptor-expressing tumor cells are complex, and required further investigations. Nevertheless, the peptide seems to induce a dual, reciprocal angiostatic and antimetastatic effect, breaking the cooperative function of cancer cells and endothelial cells during tumor progression. As shown in Fig. (**2**), DDAVP induces a tumor-mediated formation of angiostatin, a potent antiangiogenic effector [47, 50]. At the same time, the compound also activates endothelial release of VWF, which in turn obstructs biological mechanisms of cancer spread and may cause apoptosis of micrometastatic cells arrested in the target organ [49, 50, 53, 54].

Thrombosis is a well-recognized complication of cancer, particularly in several adenocarcinomas, such as of pancreas, that is associated with a higher risk of venous thromboembolism [60]. For most cancers, however, it is not clear to what extent major surgery contributes to the thrombotic risk. Chemotherapy cycles and the use of erythropoietin, thalidomide, high-dose steroids and antiangiogenic therapy increase the risk of thrombosis [61]. Although DDAVP is a safe hemostatic agent [62], the prothrombotic risk should be carefully monitored in patients administered perioperatively with the compound. DDAVP is an effective blood-saving agent for use during surgery in patients with mild hemophilia or von Willebrand disease. Antimetastatic properties of DDAVP were obtained administering intravenous doses within the range previously used to obtain antidiuretic or hemostatic effects (0.3-2 μg/kg). These doses have the advantage of being well characterized from a pharmacological point of view [35, 63].

Peptides such as DDAVP are much appreciated as lead compounds for developing human therapeutics to face the increased demand of new molecules. Pharmaceutical industries had rekindled the interest in peptides. Nowadays, more than 50 peptides are in the pharmaceutical world market, and more than a hundred are in several clinical phases. Peptides as active pharmaceutical ingredients show unique features (high biological activity, specificity and stability, and low toxicity), thereby making those attractive candidates as therapeutic agents [64, 65]. Furthermore, small peptides can be industrially prepared at rather low cost, which can fit the needs of the medical industry. Merrifield solid-phase peptide synthesis (SPPS) constitutes a key breakthrough from the perspective of accelerating research and discovery, and of its widespread application for the manufacture of peptides at the industrial scale. More than 50% of the approved peptide pharmaceuticals are manufactured using SPPS techniques [66]. Currently, a panel of linear and cyclic vasopressin peptide analogs with improved antitumor effects is in development in our laboratory [67]. Punctual amino acid substitutions in DDAVP generate novel synthetic oligopeptides with enhanced cytostatic, antimetastatic and/or antiantiogenic effects, to be tested in preclinical tumor models.

## Figures and Tables

**Fig. (1):**
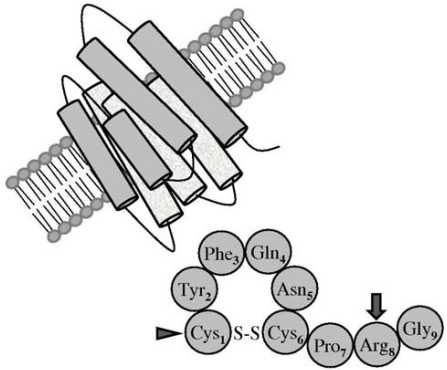
Peptide sequence of vasopressin. The synthetic analog DDAVP differs from the natural peptide by deamination of cystein in position 1 (arrowhead), which prolongs its half-life, and Darginine substitution in position 8 (arrow), which reduces the pressor effect and confers selectivity for the V2 membrane receptor.

**Fig. (2):**
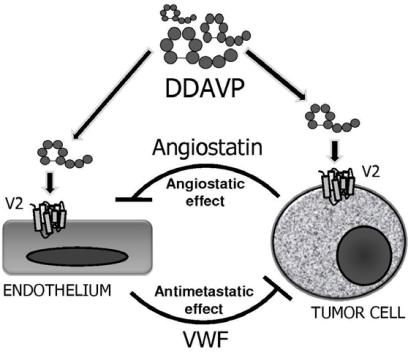
Dual and reciprocal effects of perioperative DDAVP on endothelial and tumor cells. Angiostatin is a potent angiostatic effector and VWF obstructs biological mechanisms of cancer spread and may cause apoptosis of micrometastatic foci.

**Table 1.** Blood-Saving Agents Used During Cancer Surgery

Compound	Mechanisms of Action	Type of Agent
Tranexamic acid	Inhibitor of the conversion of plasminogen to plasmin	Antifibrinolytic agent
Aprotinin	Non-specific serine protease inhibitor	Antifibrinolytic agent
DDAVP	Specific agonist of V2 vasopressin receptor on endothelial and tumor cells	Hemostatic and mild cytostatic agent

## ABBREVIATIONS

cAMP: = Cyclic adenosine monophosphate
DDAVP: = Desmopressin (1-deamino-8-D-arginine vasopressin)
ECM: = Extracellular matrix
MRD: = Minimal residual disease
RT-PCR: = Reverse transcription-polymerase chain reaction
SPSS: = Solid-phase peptide synthesis
VWF: = Von Willebrand factor
